# Unexpected winter questing activity of ticks in the Central Midwestern United States

**DOI:** 10.1371/journal.pone.0259769

**Published:** 2021-11-11

**Authors:** Ram K. Raghavan, Zoe L. Koestel, Gunavanthi Boorgula, Ali Hroobi, Roman Ganta, John Harrington, Doug Goodin, Roger W. Stich, Gary Anderson

**Affiliations:** 1 Department of Veterinary Pathobiology, College of Veterinary Medicine, University of Missouri, Columbia, Missouri, United States of America; 2 Department of Public Health, School of Health Professions, University of Missouri, Columbia, Missouri, United States of America; 3 Department of Pulmonary Immunology, Center for Biomedical Research, The University of Texas Health Science Center, Tyler, Texas, United States of America; 4 Department of Biology, College of Science, Al-Baha University, Al-Baha, Saudi Arabia; 5 Department of Diagnostic Medicine, Pathobiology, College of Veterinary Medicine, Kansas State University, Manhattan, Kansas, United States of America; 6 Independent Researcher, South Bend, WA, United States of America; 7 Kansas State Veterinary Diagnostic Laboratory, College of Veterinary Medicine, Kansas State University, Manhattan, Kansas, United States of America; University of Oklahoma Norman Campus: The University of Oklahoma, UNITED STATES

## Abstract

Unexpected questing activity of ticks was noted during the winter months of January and February in the Central Midwestern states of Kansas, Missouri, Oklahoma, and Arkansas. From nine geographically distinct locations, four species of ticks were collected using the flagging method, of which the lone star tick, *Amblyomma americanum*, was most abundant, followed by the American dog tick, *Dermacentor variabilis*, the Gulf coast tick, *Amblyomma maculatum*, and the Black legged tick, *Ixodes scapularis*. More *A*. *americanum* nymphs were caught questing than male or female adults. The winter activity of these medically important ticks in this region poses concern for public health and offers an insight into future tick activity in light of ongoing climate change. More studies on the seasonality of these tick species, and how different climate parameters affect their seasonal activity in this region are warranted and would be expected to benefit for both human and veterinary medicine.

## 1. Introduction

Tick-borne diseases in the Central Midwestern United States—roughly corresponding to the states of Kansas, Missouri, Oklahoma, and Arkansas—have been increasing steadily over the past decade [1,2]. This includes human ehrlichiosis [3], feline tularemia and cytauxzoonosis [4,5] transmitted by lone star ticks, *Amblyomma americanum*. Two novel tick-borne viral diseases of humans, caused by Heartland Virus (HRTV) and Bourbon Virus (BRBV), were recently identified in this region and were also shown to be transmitted by this tick species [6–8]. Additionally, incidences of human and animal disease agents transmitted by other tick species in the region are also on the rise. Spatiotemporal analysis of bovine anaplasmosis [9] and Rocky Mountain spotted fever [2], which are caused by rickettsial pathogens vectored by *Dermacentor variabilis*, have indicated a strong year-to-year and county-to-county spread. Increasingly also, human and canine Lyme disease cases are routinely diagnosed throughout the southeastern and eastern parts of Kansas, and recent modeling studies indicated that the vector of *Borellia burgdorferi*, Blacklegged ticks, (*Ixodes scapularis*), has likely established populations in larger swaths of land in Kansas than previously suspected [10,11]. Similar studies have shown that the potential spatial distributions of the more commonly encountered, medically-significant ticks species in this region, *A*. *americanum* [12,13], and *D*. *variabilis* [14] have expanded and/or shifted.

In the Midwestern US, and in general throughout North America, questing or host-seeking activities of these ticks (*A*. *americanum*, *D*. *variabilis*, and *I*. *scapularis*) were reported to usually occur around mid-March through mid-September, a period generally referred to as the ‘tick-season,’ with a single peak in activity occurring around the May/June period [2,15–17]. Different life stages of these tick species are seen in varying abundance throughout the tick-season. For instance, relatively more *A*. *americanum* nymphs are caught early in the season, followed by adults during mid-season and later larvae. This peak activity period is typically followed by a spike in the number of diagnosed tick-borne human disease cases, as well as canine and bovine cases during late summer and early fall. Inter-seasonal studies conducted over full year(s) in this region have been rare.

Colder temperatures limit tick activity. Even though ticks are cold-hardy and are capable of surviving through winters, they typically enter into behavioral diapause in late fall/winter and resume their host-seeking behavior only when temperatures warm up in spring [18]. Photoperiod is also an important factor that influences the onset of questing behavior among the ixodid ticks. However, tick activity may be changed during mild winters, potentially leading to higher contact rates with wildlife hosts as well as pathogen transmission to people and domestic animals [19]. To the best of our collective knowledge, winter activity of ticks is generally not reported from the Central Midwestern US. A systematic review of literature (S1 File) revealed that seventeen studies done over the past several years had documented tick collections during the winter months; however, a majority of them collected host-attached ticks, not free-living or questing ticks. Two of the 17 studies, one by Kollars and colleagues in 1999 [20] reported host attached and questing *Ixodes scapularis* ticks from southeastern Missouri, an area that is geographically/climatologically very different from the present study sites. Amblyomma and Dermacentor ticks were not found in their study during winter months. The second study is a recent work by Small and colleagues in 2019 [21], who recorded *A*. *americanum* ticks from 2 sites, from all months in a single year, and *I*. *scapularis* from only the winter months in Oklahoma County, in Oklahoma. Of the 2,019 ticks they collected, over 95% were captured in CO_2_ traps. CO_2_ traps have been shown to result in different capture rate compared to cloth drags [22]. Unlike CO_2_ traps, which act as an attractant kairomone for ticks, the dragging method almost exclusively captures ticks that are already questing without an external stimulus other than higher ambient temperature and any host-related stimulus in the environment. For this reason, drag sampling may be reveal relatively more of the risk for human and animal contact with ticks.

In this study, we asked if different tick species in this region were actively questing through the winter of 2017, a relatively mild winter due to the *La Nina* effect, with the aim being to better assess risk of transmission of tick-borne disease agents.

## 2. Materials and methods

**Fig 1:**
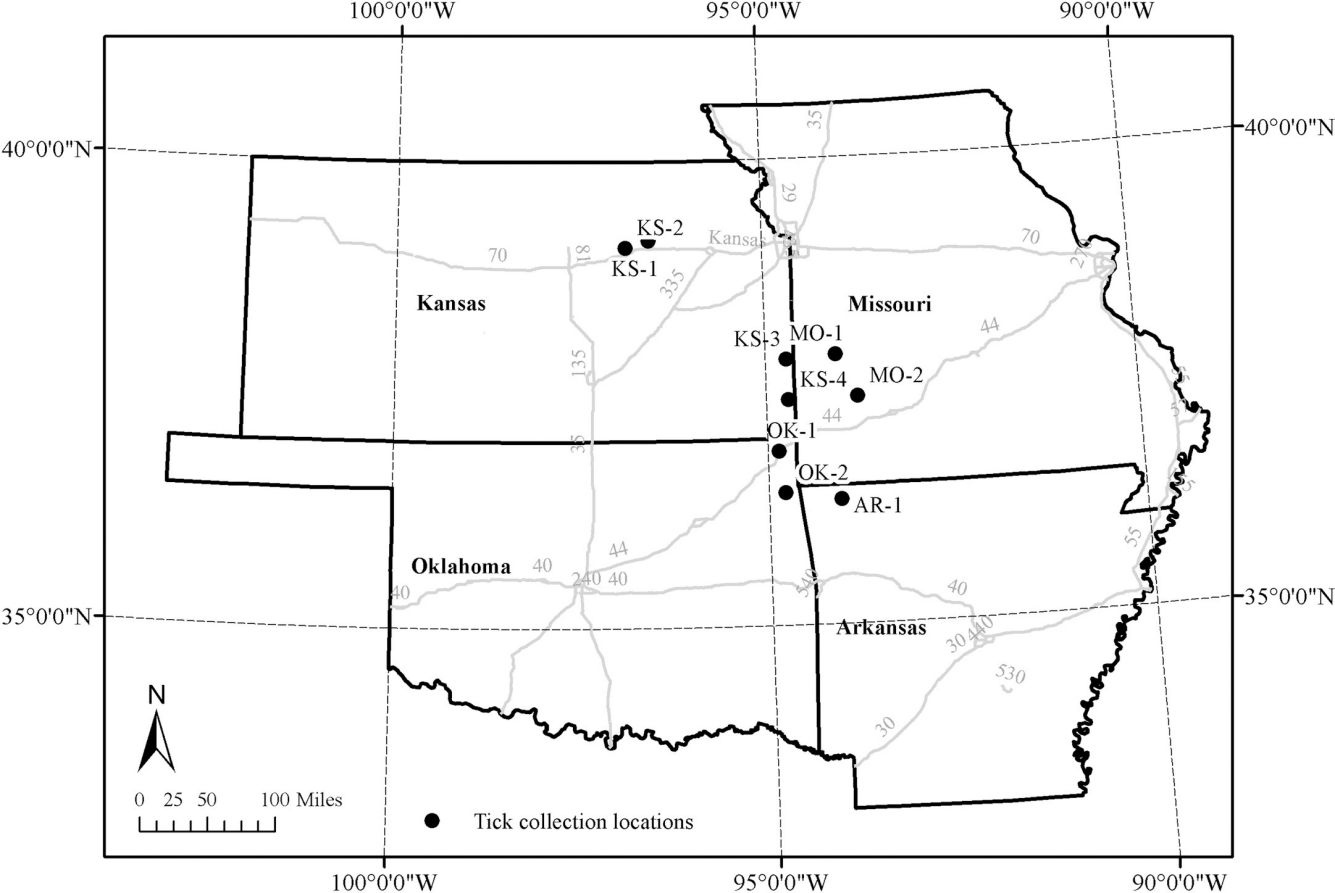
Locations where questing ticks were collected during the winter months of January and February of 2017.

Ticks were collected from nine geographically distinct locations, four of which were situated in Kansas and the remaining sites were in Oklahoma (2), Arkansas (1) and Missouri (2) (Fig 1). These sites had varying land cover/land use characteristics ranging from predominantly prairie/grassland (3), wooded areas (2), agricultural land and/or pasture (2), and areas used by the public for outdoor recreational activities that had a mixture of wooded and meadow land cover types (2). All the collection sites had confirmed tick presence during our previous collection efforts in prior years (2015, 2016) during the regular tick season.

Ticks were collected from each location using the flagging method [23] by two persons for 1 hour within a 3 hour time-window, between 11:30 AM to 2:30 PM. Collections were conducted whenever outside temperatures were above 0°C. Areas covered under snow or melting ice were avoided, and flagging was done following a grid pattern over vegetation. The 1 m^2^ flannel cloths, used for flagging were replaced with newer ones when they became unusable, mostly due to getting wet and/or soiled. All sites were visited randomly twice in the month of January and twice in February, with one week separating collection events. Winter months in the study region occur between mid-December through mid-March.

Temperature and relative humidity conditions at the time of collection were recorded using a Hobo^®^ data loggers (Model: U23 Pro v2; Onset Computer Corporation, Bourne, MA) at approximately 1 meter above the ground level, on an even surface away from direct sunlight. Ticks were transported to the laboratory on ice and identified under a microscope using taxonomic keys to differentiate species, sex, and development stage.

Rootograms were used to determine the appropriate statistical distribution for the observed count data. Following this, Generalized Negative Binomial mixed two-way ANOVA using R package lme4 with species as between and weeks as within factor were used to test any overall difference in the average counts between species, weeks and species-weeks combinations [24,25]. Tukey’s post-hoc analysis was used to further investigate the difference for significant effects. Separately, chi-square tests for independence were used to assess if there were any associations in the number of ticks (species or life-stage) with the land cover type. For each species, independence between sex/life stage and land cover was also assessed.

## 3. Results

**Fig 2:**
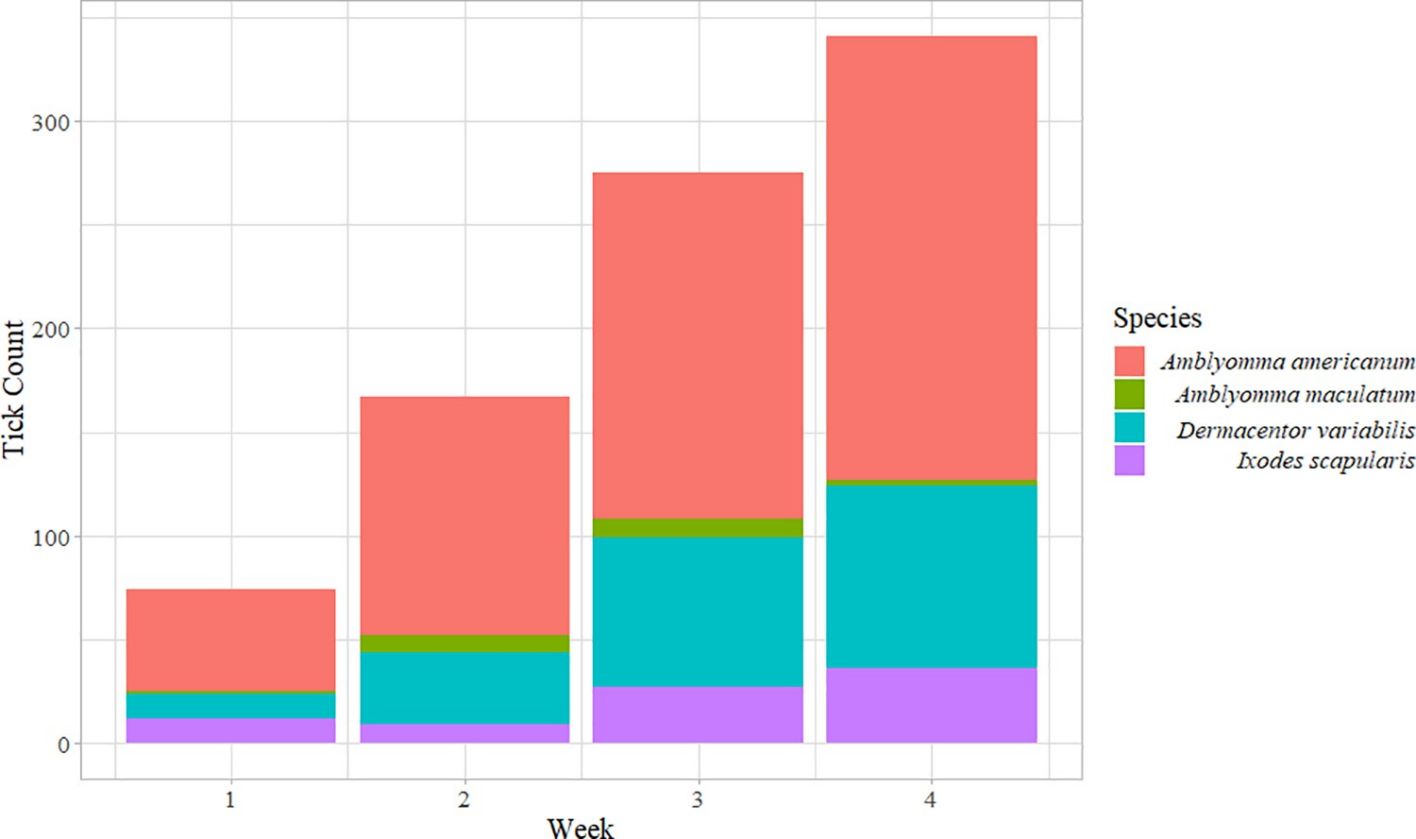
Ticks collected from the study region broken down by each week.

**Fig 3:**
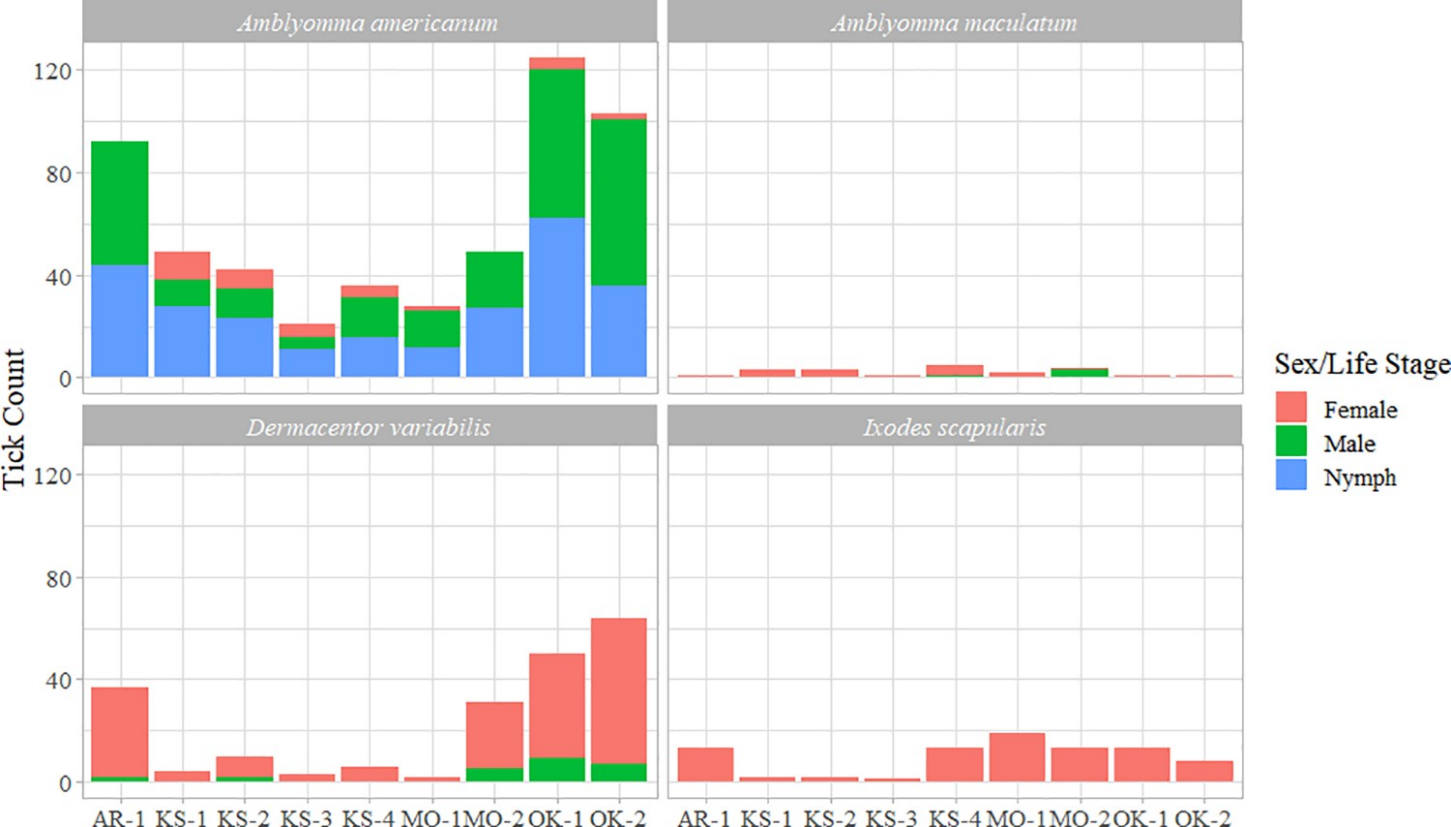
Ticks collected from the study region broken down by individual locations.

Four questing tick species, *A*. *americanum*, *A*. *maculatum*, *D*. *variabilis* and *I*. *scapularis* were caught in this study during the winter months of 2017. Of these, *A*. *americanum* ticks were the most abundant (n = 545) followed by *D*. *variabilis* (n = 207), *I*. *scapularis* (n = 84) and *A*. *maculatum* (n = 21). Among *A*. *americanum* ticks, more nymphs were caught followed by male and female adults. Over the course of the two-month period, total 259 nymphal, 191 male and 95 female *A*. *americanum*; and 61 male and 146 female *D*. *variabilis*, as well as 19 male and 65 female *I*. *scapularis* and 7 female and 14 male *A*. *maculatum* were caught (Tables 1–4), Figs 2 and 3, S1 Table**)**.

**Table 1:** Number of ticks caught in different locations during January 3 –January 7, 2017 and the ambient temperature and relative humidity.

Location	Date	Temperature °C	Relative humidity (%)	*A*. *americanum* (n)			*A*. *maculatum* (n)		*D*. *variabilis* (n)		*I*. *scapularis* (n)	
				Nymph	M	F	M	F	M	F	M	F
KS-1	Jan 3	2.77	65	1	0	0	0	0	1	1	0	0
KS-2	Jan 3	3.20	70	0	0	0	0	0	1	0	0	0
KS-3	Jan 4	2.81	75	0	0	0	0	0	0	0	0	1
KS-4	Jan 4	4.22	81	7	8	1	0	0	0	1	1	1
MO-1	Jan 5	4.61	68	4	4	1	0	0	0	0	0	2
MO-2	Jan 6	-6.52	70	-	-	-	-	-	-	-	-	-
AR-1	Jan 6	-5.80	61	-	-	-	-	-	-	-	-	-
OK-1	Jan 7	6.61	71	8	5	3	0	0	1	3	1	3
OK-2	Jan 7	8.10	68	4	2	1	1	0	0	4	0	3

**Table 2:** Number of ticks caught in different locations during January 16 –January 20, 2017 and the ambient temperature and relative humidity.

Location	Date	Temperature °C	Relative humidity (%)	*A*. *americanum* (n)			*A*. *maculatum* (n)		*D*. *variabilis* (n)		*I*. *scapularis* (n)	
				Nymph	M	F	M	F	M	F	M	F
KS-1	Jan 16	13.33	62	4	1	2	0	1	0	2	0	1
KS-2	Jan 16	4.25	81	6	2	1	0	0	0	0	0	0
KS-3	Jan 17	5.61	84	0	1	0	0	0	0	0	0	0
KS-4	Jan 17	7.21	95	4	0	4	0	2	0	0	0	3
MO-1	Jan 17	7.21	67	6	2	1	1	1	0	1	0	4
MO-2	Jan 18	7.64	70	4	0	1	0	1	0	3	1	0
AR-1	Jan 28	10.13	90	9	7	2	1	0	1	3	0	0
OK-1	Jan 19	12.77	94	15	11	7	0	1	3	8	0	0
OK-2	Jan 20	20.10	70	12	8	5	0	0	5	9	0	0

(n) = number of live ticks collected.

**Table 3:** Number of ticks caught in different locations during January 30 –February 3, 2017 and the ambient temperature and relative humidity.

Location	Date	Temperature °C	Relative humidity (%)	*A*. *americanum* (n)			*A*. *maculatum* (n)		*D*. *variabilis* (n)		*I*. *scapularis* (n)	
				Nymph	M	F	M	F	M	F	M	F
KS-1	Jan 30	18.88	54	11	1	2	0	2	0	2	0	1
KS-2	Jan 30	18.59	99	8	1	3	1	0	0	1	0	0
KS-3	Jan 31	7.22	58	3	0	1	0	1	0	1	0	0
KS-4	Jan 31	14.44	70	4	3	1	1	1	1	2	0	3
MO-1	Feb 1	11.6	64	0	4	1	0	0	0	0	1	4
MO-2	Feb 2	12.4	54	15	7	1	1	2	5	11	2	3
AR-1	Feb 2	8.91	60	17	18	2	0	0	3	13	1	5
OK-1	Feb 3	3.88	52	18	12	1	0	0	5	8	1	3
OK-2	Feb 3	5.74	54	3	23	7	0	0	3	17	2	1

(n) = number of live ticks collected.

**Table 4:** Number of ticks caught in different locations during January 13 –January 17, 2017 and the ambient temperature and relative humidity.

Location	Date	Temperature °C	Relative humidity (%)	*A*. *americanum* (n)			*A*. *maculatum* (n)		*D*. *variabilis* (n)		*I*. *scapularis* (n)	
				Nymph	M	F	M	F	M	F	M	F
KS-1	Feb 13	10.55	57	12	8	7	0	0	0	0	0	0
KS-2	Feb 13	20.55	73	9	7	5	1	1	0	7	0	2
KS-3	Feb 14	7.77	71	8	4	4	0	0	0	1	0	0
KS-4	Feb 14	11.11	70	1	0	3	0	1	1	1	1	4
MO-1	Feb 15	12.2	62	2	0	3	0	0	0	1	2	6
MO-2	Feb 15	12.8	75	8	9	4	0	0	3	9	2	5
AR-1	Feb 16	17.3	61	18	11	8	0	0	7	10	1	6
OK-1	Feb 17	20.55	54	21	17	7	0	0	9	13	2	3
OK-2	Feb 17	18.52	74	17	15	6	0	0	12	14	1	1

(n) = number of live ticks collected.

**Fig 4:**
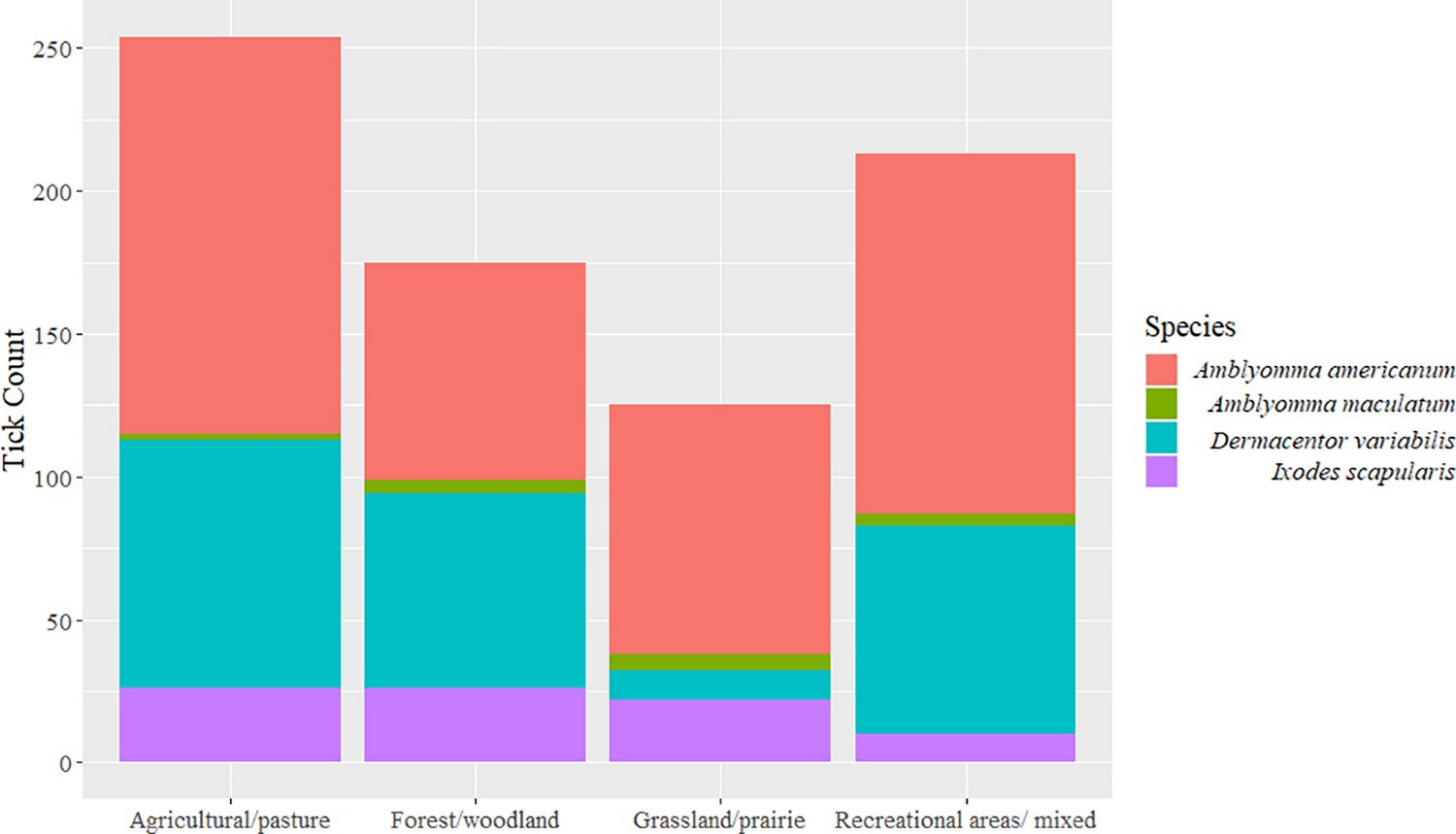
Ticks collected from the study region broken down by different landcover types.

The number of ticks collected from each location was variable among sites of different landcover characteristics (Fig 4). Sites with predominantly forest/woodland land cover and recreational areas with mixed land cover types consistently yielded more ticks of all four species (Table 5). Relatively large clusters of *D*. *variabilis* nymphs were collected in three instances from some sites during the collection effort. No larval ticks of any of the species were observed in the collections during study. In general, more *A*. *americanum* and *D*. *variabilis* ticks from all sites were caught as the study progressed, and it appeared that more ticks were actively questing during the relatively warmer days. Temperatures at collection sites ranged from 2.77°C earlier in the study to 20.55°C towards the end. Higher temperatures were recorded in the southern extent of the study region, in sites bordering the states of Kansas, Oklahoma, and Arkansas. Relative humidity during the study ranged from 52–90% and there were no recognizable spatial or temporal trend.

**Table 5:** Number of ticks caught in different land cover/land use types.

Land cover/land use	Grassland/prairie [KS-1, KS-3, MO-1] (n)	Forest/woodland [AR-1, MO-2] (n)	Agricultural/pasture [AR-1, OK-1] (n)	Recreational areas/ mixed [KS-2, OK-2] (n)
***Amblyomma americanum***				
Nymph	40	6	28	40
Male	25	52	81	58
Female	22	18	30	28
***Dermacentor variabilis***				
Male	1	19	29	21
Female	9	49	58	52
***Amblyomma maculatum***				
Male	1	2	1	3
Female	5	3	1	1
***Ixodes scapularis***				
Male	3	7	6	3
Female	19	19	20	7

(n) = number of live ticks collected.

The Rootogram plots revealed that a negative-binomial distribution better described the observed count data for ticks across weeks and different landcover types. The Generalized Negative Binomial mixed two-way ANOVA revealed significant difference between species (χ^2^ *= 22*.*003*, *DF = 3*, *P = 6*.*514e-05*) and between weeks ((χ^2^ = 15.551, DF = 3, *P* = 0.001) overall and a Tukey’s test further revealed that the number of tick species collected each week was significantly different from one another. No interaction effect between species and weeks were however noted (χ^2^ *= 10*.*602*, *DF = 9*, *P = 0*.*304*). For all weeks, *A*. *americanum* had the greatest count, followed by *D*. *variabilis*, *I*. *scapularis*, and *A*. *maculatum*. From week 2 to week 3, there was an increase in log counts of species (*mean difference = -0*.*573*, *SE = 0*.*222*, *Z = -2*.*584*, *P = 0*.*048*), but this increase was similar in magnitude for all species (Fig 2). The Chi-square test for independence revealed significant association between the sex/life-stage of *A*. *americanum* and land cover (χ^2^ *= 41*.*208*, *DF = 6*, *P = 2*.*63*^*−07*^); however, none of the other species independently showed a similar association with landcover type [*D*. *variabilis* (*Chisq = 2*.*545*, *DF = 3*, *P = 0*.*467*), *A*. *maculatum* (*D*. *variabilis* (χ^2^ *= 2*.*545*, *DF = 3*, *P = 0*.*467*), *A*. *maculatum* (χ^2^ *= 3*.*445*, *DF = 3*, *P = 0*.*328*), *I*. *scapularis* (χ^2^ *= 1*.*604*, *DF = 3*, *P = 0*.*659*)].

Collection attempts were aborted for two sites, during the first week of this study due to temperatures falling below 0°C.

## 4. Discussion

Winter activity of questing ixodid ticks has been previously reported elsewhere [18,19]. Knowledge of this matter is important for an accurate understanding of tick seasonal activities, mainly due to the public health and animal health relevance, and for understanding how climate and ecological forces shape their spatiotemporal distribution. Although some have reported minimal activity of *A*. *americanum* during winter months in regions south of the current study [26,27] most studies that report tick seasonality in the region have considered the traditional tick season for their studies [16,28–30] and do not include collections during the winter, and it often has been generally assumed that *A*. *americanum* and *D*. *variabilis* ticks are not active during the winter months. To the best of our knowledge, records indicating the detection of *I*. *scapularis* or *A*. *maculatum* in Kansas, even during the regular tick season, within the past decade cannot be seen in published literature (S1 File). The discovery of four tick species in relatively high numbers, actively seeking hosts in the middle of winter, changes some of these perceptions. It is worth noting that ticks of all four species mentioned were collected even when ambient temperatures at collection sites were below 10°C (but > 0°C). Early in the study, *A*. *americanum* and *D*. *variabilis* ticks were caught when the air temperature roughly 1 meter above ground was a mere 2.77°C, the lowest level recorded in the study.

### SF 2. Literature search of tick winter activity in the Midwestern USA

Tick collections made during regular tick-seasons (*i*.*e*., mid-March through mid-September) in this region using the flagging or dragging methods primarily yield *A*. *americanum* and *D*. *variabilis* ticks [29]. Over the past five-year period, we have also consistently collected *A*. *maculatum* and *I*. *scapularis* in the same region, albeit in relatively low numbers. This tick community composition is identical to the composition in the present study, indicating that the composition does not change over the course of a year, and that all tick species actively quest when conditions are favorable, even during winter. A notable difference in the present study, however, is the higher relative abundance of nymphal ticks caught compared to their adult counterparts. It is likely that the nymphs did not go into behavioral diapause or went into diapause late in the fall/onset of winter but became active when conditions were suitable or spring-like. Unlike collections conducted during regular tick-season, we did not find larval ticks in this attempt, which corroborated previous reports of collections from deer [26,27] and cattle [27] and could be indicative of their avoidance of colder conditions due to different physiological requirements. Also, there were more females collected than males in all the species. This finding is consistent with our previous observations during regular collections in where female bias was observed.

There were notable differences in the relative abundance of *A*. *americanum* and *D*. *variabilis* ticks caught in sites with different land cover/land use types. The GLM and analysis of variance, as well as chi-square tests for independence revealed significant weekly patterns in the log counts of ticks collected, and a significant association between land cover and *A*. *americanum* counts. However, more robust statistical comparisons could not be made due to the relatively short duration over which the study was conducted. Our objective in this study was purely to observe and qualitatively assess if any questing activity whatsoever could be seen during winter months. More robust studies to quantify land cover/land use influences on winter questing activity of these ticks may yield useful insights into their host-seeking adaptive strategies and provide further material for informed public health messaging. In fact, our earlier work involving some of the same collection sites that were included in this study indicated that tick presence and further tick-borne pathogen prevalence is influenced by land cover type and the physical properties of land cover such as fragmentation [2].

Globally, the conservative climate models used by the Intergovernmental Panel on Climate Change (IPCC) project a 0.2°C rise in temperatures per decade for the next two decades worldwide [31]. At the current rate of greenhouse gas emissions, an increase of up to 4° C by the end of the century is projected. Recent climate trends in the northern latitudes generally indicate a warming pattern over the winter months as well as more humid summers [32]. Due to climate change, temperatures in Kansas are expected to increase by 0.4°C per decade in summer and fall and precipitation will decrease in summer and fall and increase slightly in winter [33]. Such changes in climate could have profound effects on tick ecology and disease epidemiology. For two consecutive winters, 2015–16 and 2016–17, warmer temperatures were experienced in the Central Midwestern US [34]. This warming occurred with opposite phases of ENSO (*El Nino* in 2015–16 and *La Nina* in 2016–17), reinforcing the effect of climate-change rather than extreme conditions in the tropical Pacific. These two recent warm winters provide an opportunity for us to understand how relatively long-term temperature and humidity changes due to climate change may influence tick activity under natural settings.

Climate is the primary limiting factor that affects the spatiotemporal distribution and abundance of ticks because some tick species do not survive accumulative cold temperatures below a certain threshold [35]. Additionally, tick behavioral diapause that affects their seasonality is largely determined by photoperiod [18,19]. At present, it is unclear what those cold (or hot) thresholds are for the four tick species in this study region, and, the phenology of these tick species are only beginning to be studied in detail [36]. Temporal overlapping of tick life-stage cohorts with one another plays a large role in how pathogens are transferred among themselves and with their wildlife hosts, another aspect of ecology that requires further study for the four tick species in this region. Warmer winters with only a few days below any developmental or behavioral thresholds will allow ticks to increase in abundance, extend their questing period, and may help to establish populations in new areas [2]. Other less discussed effects are on the levels of tick-borne pathogens, such as favorable survival and the emergence of novel tick-borne pathogens, which need to be assessed.

## Conclusions

With the ongoing climate change, it can be expected that the seasonal activity of medically significant tick species in the central midwestern US will change, with these tick species becoming more active throughout the year. With a change in seasonality, the epidemiology of diseases caused by agents transmitted by these tick species will also change, likely increasing beyond the current incidence levels. The central midwestern US has seen a steady surge in diagnosed tick-borne diseases over the past decade, a trend that is consistent with other regions in the US. Also, two novel tick-borne viruses were recently identified in the central Midwest, which have led to deaths of individuals. Urgent studies are needed to identify the seasonality of tick species and pathogen prevalence, and large-scale/micro-scale mechanisms involved in the regulation of tick life cycles so that the effects of climate change can be better understood and accurate, effective public health messaging can be executed.

## Supporting information

S1 Table Tabular data of ticks collected at different sites during the study. (XLSX)

S1 File Literature search of tick winter activity in the Midwestern USA. (DOCX)
