# Supplementary material for: Unexpected winter questing activity of ticks in the Central Midwestern United States
Source: PLoS One. 2021 Nov 11;16(11):e0259769. doi: 10.1371/journal.pone.0259769 (PMC8584693; doi:10.1371/journal.pone.0259769)
Supplement: S1 File — (DOCX) [file pone.0259769.s002.docx]

**S1 File.**

Literature search:

On a systematic survey of literature; PubMed, Scopus, Web of Science (BIOSIS Citation Index) and CAB with the following keywords:

"Kansas" OR “Oklahoma” OR “Arkansas” OR “Missouri” OR “Midwestern United States” OR “Midwest”) AND ("winter" OR “season*” OR “climate change” OR “January” OR “February”) AND (“*Amblyomma americanum*” OR “*Dermacentor variabilis*” OR “*Amblyomma maculatum*” OR “*Ixodes scapularis*”)

171 articles were retrieved, of which 101 were unique. From these, publications were queried for further relevance; particularly, collection of ticks in the winter months. This resulted in 17 articles, including the two studies mentioned above. The other studies predominantly collected host-attached ticks during winter months, not questing ticks. Results of literature survey of tick phenology in the Central Midwestern US are listed below.

1. Barker, R. W., Kocan, A. A., Ewing, S. A., Wettemann, R. P., & Payton, M. E. (2004, March). Occurrence of the Gulf Coast tick (Acari: Ixodidae) on wild and domestic mammals in north-central Oklahoma . Journal of Medical Entomology, 41(2), 170-178. https://doi.org/10.1603/0022-2585-41.2.170
2. Barnard, D. R. (1981a). *Amblyomma americanum*: comparison of populations of ticks free living on pasture and parasitic on cattle. Annals of the Entomological Society of America, 74(5), 507-511. https://doi.org/10.1093/aesa/74.5.507
3. Barnard, D. R. (1981b). Seasonal activity and preferred attachment sites of Ixodes scapularis (Acari: Ixodidae) on cattle in southeastern Oklahoma. Journal of the Kansas Entomological Society, 54(3), 547-552.
4. Barnard, D. R., Morrison, R. D., & Popham, T. W. (1985). Light and temperature sensitivity of feeding-related and reproductive processes in *Amblyomma americanum* (Acari: Ixodidae) on cattle. Environmental Entomology, 14(4), 479-485. https://doi.org/10.1093/ee/14.4.479
5. Bouzek, D. C., Fore, S. A., Bevell, J. G., & Kim, H. J. (2013, Jun). A conceptual model of the Amblyomma americanum life cycle in northeast Missouri . Journal of Vector Ecology, 38(1), 74-81. https://doi.org/10.1111/j.1948-7134.2013.12011.x
6. Fortman, R. W. (1944). Winter distribution of two ectoparasites of the cottontail rabbit in Missouri. Journal of Economic Entomology, 37, 541. https://doi.org/10.1093/jee/37.4.541
7. Gage, K. L., Hopla, C. E., & Schwan, T. G. (1992, Sep). Cotton rats and other small mammals as hosts for immature Dermacentor variabilis (Acari: Ixodidae) in central Oklahoma. J Med Entomol, 29(5), 832-842. https://doi.org/10.1093/jmedent/29.5.832
8. Garvin, S. D., Noden, B. H., Dillwith, J. W., Fox, S. F., Payton, M. E., & Barker, R. W. (2015, Sep). Sylvatic Infestation of Oklahoma Reptiles with Immature Ixodes scapularis (Acari: Ixodidae) . Journal of Medical Entomology, 52(5), 873-878. https://doi.org/10.1093/jme/tjv100
9. Koch, H. G. (1982). Seasonal incidence and attachment sites of ticks (Acari: Ixodidae) on domestic dogs in southeastern Oklahoma and northwestern Arkansas USA. . Journal of Medical Entomology, 19(3), 293-298. https://doi.org/10.1093/jmedent/19.3.293
10. Kollars, T. M., Jr., Oliver, J. H., Jr., Durden, L. A., & Kollars, P. G. (2000, October). Host associations and seasonal activity of Amblyomma americanum (Acari: Ixodidae) in Missouri . Journal of Parasitology, 86(5), 1156-1159.
11. Kollars, T. M., Jr., Oliver, J. H., Jr., Kollars, P. G., & Durden, L. A. (1999, Nov.). Seasonal activity and host associations of *Ixodes scapularis* (Acari: Ixodidae) in southeastern Missouri . Journal of Medical Entomology, 36(6), 720-726. https://doi.org/10.1093/jmedent/36.6.720
12. Kollars, T. M., Jr., Oliver, J. H., Jr., Masters, E. J., Kollars, P. G., & Durden, L. A. (2000). Host utilization and seasonal occurrence of Dermacentor species (Acari: Ixodidae) in Missouri, USA . Experimental and Applied Acarology, 24(8), 631-643. https://doi.org/10.1023/a:1026566301325
13. Mangan, M. J., Foré, S. A., & Kim, H. J. (2018, Dec). Ecological modeling over seven years to describe the number of host-seeking *Amblyomma americanum* in each life stage in northeast Missouri. J Vector Ecol, 43(2), 271-284. https://doi.org/10.1111/jvec.12311
14. Patrick, C. D., & Hair, J. A. (1977). Seasonal abundance of lone star ticks on white-tailed deer. Environmental Entomology, 6(2), 263-269. https://doi.org/10.1093/ee/6.2.263
15. Portman, R. W., & Dalke, P. D. (1945). Infestation of a Red Fox by *Ambylomma americanum*. Journal of Economic Entomology, 38(3), 397 p. https://doi.org/10.1093/jee/38.3.397
16. Semtner, P. J., & Hair, J. A. (1973). DISTRIBUTION SEASONAL ABUNDANCE AND HOSTS OF THE GULF COAST TICK . Annals of the Entomological Society of America, 66(6), 1264-1268. https://doi.org/10.1093/aesa/66.6.1264
17. Small, M. M., Laverty, S. M., King, C. B., & Brennan, R. E. (2019, Jun). Tick species establishment in Oklahoma County, Oklahoma, USA, identified by seasonal sampling in residential and non-residential sites . Journal of Vector Ecology, 44(1), 105-111. https://doi.org/10.1111/jvec.12334
